# Whole-Body Reactive Agility Metrics to Identify Football Players With a Core and Lower Extremity Injury Risk

**DOI:** 10.3389/fspor.2021.733567

**Published:** 2021-10-20

**Authors:** Scott L. Bruce, Gary B. Wilkerson

**Affiliations:** ^1^Masters of Athletic Training Program, Arkansas State University, Jonesboro, AR, United States; ^2^Department of Health and Human Performance, University of Tennessee at Chattanooga, Chattanooga, TN, United States

**Keywords:** performance assessment, prediction modeling, asymmetry, whole-body reactive agility, reaction time, speed, acceleration, deceleration

## Abstract

Clinical prediction models are useful in addressing several orthopedic conditions with various cohorts. American football provides a good population for attempting to predict injuries due to their relatively high injury rate. Physical performance can be assessed a variety of ways using an assortment of different tests to assess a diverse set of metrics, which may include reaction time, speed, acceleration, and deceleration. Asymmetry, the difference between right and left performance has been identified as a possible risk factor for injury. The purpose of this study was to determine the whole-body reactive agility metrics that would identify Division I football players who were at elevated risk for core, and lower extremity injuries (CLEI). This cohort study utilized 177 Division I football players with a total of 57 CLEI suffered who were baseline tested prior to the season. Single-task and dual-task whole-body reactive agility movements in lateral and diagonal direction reacting to virtual reality targets were analyzed separately. Receiver operator characteristic (ROC) analyses narrowed the 34 original predictor variables to five variables. Logistic regression analysis determined the three strongest predictors of CLEI for this cohort to be: lateral agility acceleration asymmetry, lateral flanker deceleration asymmetry, and diagonal agility reaction time average. Univariable analysis found odds ratios to range from 1.98 to 2.75 for these predictors of CLEI. ROC analysis had an area under the curve of 0.702 for any combination of two or more risk factors produced an odds ratio of 5.5 for risk of CLEI. These results suggest an asymmetry of 8–15% on two of the identified metrics or a slowed reaction time of ≥0.787 s places someone at increased risk of injury. Sixty-three percent (36/57) of the players who sustained an injury had ≥2 positive predictors In spite of the recognized limitation, these finding support the belief that whole-body reactive agility performance can identify Division I football players who are at elevated risk for CLEI.

## Introduction

Clinical prediction models have been created for a variety of orthopedic conditions using a variety of cohorts (Gribble et al., 2016; Moran et al., 2017; Panken et al., 2017; Beltran-Alacreu et al., 2018; Bruce et al., 2018; De Blaiser et al., 2018; Frangiamore et al., 2020). A useful clinical prediction model must go through three phases of development and evaluation. The first step is to establish a strong relationship between the predictor variables and the injury or injuries being examined. Step two is the validation of a prediction model on a different, yet similar cohort. The third step is evaluating the model on clinical practice and whether or not a positive change occurs. The primary problems in the application of prediction models to similar cohorts are differences in the predictor variables, or the metrics assessed, or minor deviations in how performance tests are administered. To date, no universal screening tests are available to predict musculoskeletal injuries as the risk factors are highly population-specific. Additionally, there is a lack of intervention studies to provide sufficient support for specific injury risk screening (Bahr, 2016).

American football offers a good population and setting for attempting to predict injuries. Typical college football teams have about 100 or more players on each team. At the intercollegiate level, the injury rate for football is the second highest, trailing only wrestling [7.29–9.28 injuries per 1,000 Athlete-Exposures (A-Es)]. Although the number of players on a high school football teams are considerably less than intercollegiate teams and are highly dependent upon the size of the school, high school football has the highest injury rate of any sport with 4.01 injuries per 1,000 A-Es. High school wrestling has the next highest injury rate at 2.38 injuries per 1,000 A-Es (Kerr et al., 2018; Kroshus et al., 2018). If examining other sports, then either a large number of teams have to be recruited for the assessment or a school's team needs to be followed for several years. For this study, we used intercollegiate football players.

There are many types of physical performance assessments, which athletes are asked to perform in an effort to quantify their neuromuscular performance capabilities. Tests that involve lower extremity movement patterns such as: jump, hop, balance and reach, excursion, lunge, and step-up/down have been utilized (Huxel Bliven and Anderson, 2013; Park et al., 2013; McGovern et al., 2018; Bagherian et al., 2019). Additionally, tests involving sprints, shuttle runs, bench presses, push-up tests, and core muscular endurance tests have also been used to assess functional movements (Hegedus et al., 2015; Nuzzo, 2015; Tarara et al., 2016; Bruce et al., 2017). These test variables are measurements for time, for distance, for height, or for repetitions. The main problem with many of these tests is they tend to focus on a single performance factor and are not activities that closely replicate American football-specific movement patterns.

Measuring multiple capabilities during execution of sport-specific movements with simultaneous imposition of cognitive demand would be optimal. Whole-body reactive agility (WBRA) involves integration of perceptual, cognitive, and motor processes that are required in athletic activities (Wilkerson et al., 2018; Araújo et al., 2019). Metrics such as reaction time (RT), speed, acceleration and deceleration () reflect performance capabilities required for both athletic success and injury avoidance, but simultaneous assessment can be difficult (De Blaiser et al., 2018; Vereijken et al., 2020).

Another problem is identification of the most important movement variables. Neuromechanical responsiveness is the ability to integrate neurocognitive and neuromechanical processes during athletic activities (Wilkerson et al., 2018). Intuitively, athletes with the quickest reaction time, and the fastest speed, acceleration and deceleration would be the better performers. Likewise, when two athletes or two teams are mismatched it is generally easy to tell which person or team is faster. The problem occurs when assessing a group of athletes who are similar in many of these metrics. A single assessment session does not provide for all aspects of the complex systems involved in functional movement. Factors such as time, motivation, energy levels, stress, emotions, pain or psychosocial factors cannot be accounted for in a single session (Fonseca et al., 2020). Multiple testing sessions will permit a more complete picture of the athlete's neuromechanical responsiveness.

Reaction time is fundamental to sports activity and everyday life. Athletes must react to other players or to the ball. Drivers must step on a brake or turn the steering wheel to avoid a crash. Walking or moving requires one to react to ground reaction forces or environmental stimuli to avoid a fall or injury (Lempke et al., 2020). There are several types of RT based on the stimulus and the resulting response. Simple RT is the quickest response to a stimulus with little to no conscious thought. Choice RT involves a go-no go decision to be made in response to the stimulus. Discrimination RT entails responding to a complex stimulus to include a more extensive cognitive process causing a decision between the correct responses over an incorrect response. This increase in cognitive involvement prolongs the completion of the response (Jensen, 2006). Regardless of the type of RT it consists of two parts: latency time, and motor time. Latency time is the time from the appearance of the stimulus to the initiation of the movement response. Motor time is the time from the initiation of the movement to completing the response to the stimulus (Gallagher et al., 2020).

Through the years, the value coaches place on speed has been well-documented in the sports media. However, the ability to accelerate and decelerate may be bigger keys in athletics. American football has shown higher intensities in accelerations compared to decelerations. All other sports had higher decelerations than accelerations (Harper et al., 2019).

A variable that should be considered in any injury prediction model is performance asymmetry. An asymmetry (Asym) represents differences between right and left direction and is expressed as a percentage (Gordon et al., 2021). Helme et al., did a systematic review of 14 clinical prediction model studies with half of the studies finding statistically significant results. Asymmetries were assessed with the functional movement screening in three of the seven statistically significant studies, two utilized the Star Excursion Balance test, and one examined the Y-balance test. The seventh study utilized isokinetic testing. Six of the seven studies reported their findings with positive likelihood ratios that ranged from 1.78 to 2.72 (mean = 2.15, sd ± 0.402) (Helme et al., 2021). Whole-body reactive agility Asyms represent differences in movement performance capabilities since both extremities contribute to the movement of the body in any direction (Wilkerson et al., 2020).

Single-task activities involves the player performing either a cognitive task or a movement task in isolation. Dual-task activities require an athlete to cognitively process a variety of sensory inputs, and then produce a correct physical response. Cognitive demand imposed during movement defines a dual-task activity (Büttner et al., 2020; Wingerson et al., 2020). Athletic activities require perception of changing environmental conditions, decision-making, and activation of effective motor patterns to either avoid collisions or resist potentially injurious external forces (Giza and Hovda, 2014; Büttner et al., 2020; Ness et al., 2020). Many sport-specific tasks require dual-task neural processing (Ness et al., 2020), but most clinical assessments involve single-task activities.

Core and lower extremity injuries (CLEI) have been studied previously in prediction models (Wilkerson et al., 2012; Wilkerson and Colston, 2015). Research has demonstrated that poor core stability is related to low back pain. Low back pain has been associated with lower extremity injury, and lower extremity injury predisposes one having low back pain (Leetun et al., 2004; Hart et al., 2006; Zazulak et al., 2007; Hammill et al., 2008; Wilkerson et al., 2012; Wilkerson and Colston, 2015). For these reasons we focused our analysis on CLEI.

Although previous studies have examined components of WBRA such as reaction time, speed, acceleration, deceleration, asymmetries, and dual-task metrics, only a few have examined these variables jointly (Wilkerson et al., 2017, 2018, 2020, 2021a; Hogg et al., 2021). None of these studies examined these metrics specifically for CLEI in intercollegiate football players. Therefore, our purpose was to determine the WBRA metrics that would identify Division I football players who were at elevated risk for CLEI. Our research hypothesis was we would be able to create clinical prediction model using WMBR metrics to identify Division I football players with increased risk of CLEI.

## Methods

### Participants

This study utilized a cohort of two different NCAA Division I football programs (*n* = 177): Team A was a Football Bowl Subdivision (FBS) team (*n* = 102); Team B was a Football Championship Subdivision (FCS) program (*n* = 75). Each player was tested prior to the beginning of pre-season camp in 2019 and 2020, for WBRA. Testing was part of the evaluation process utilized by the strength and conditioning staff at each university. In cases where football players were tested for both seasons, a player's initial assessment was used in the analysis and corresponding injury data for that season were used.

### Procedures

**Figure 1:**
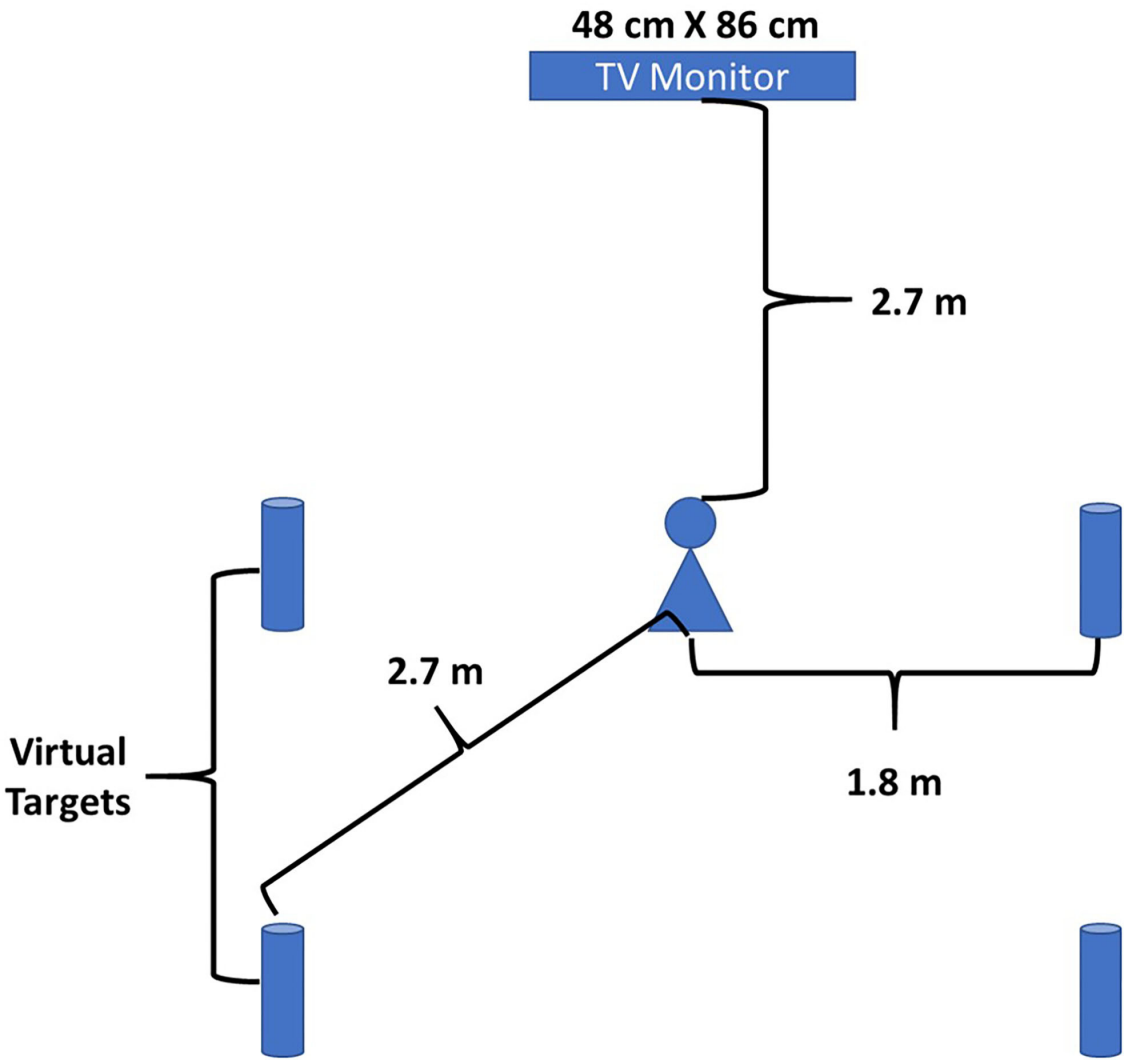
Schematic of the set-up used for the WBRA tests.

**Figure 2:**
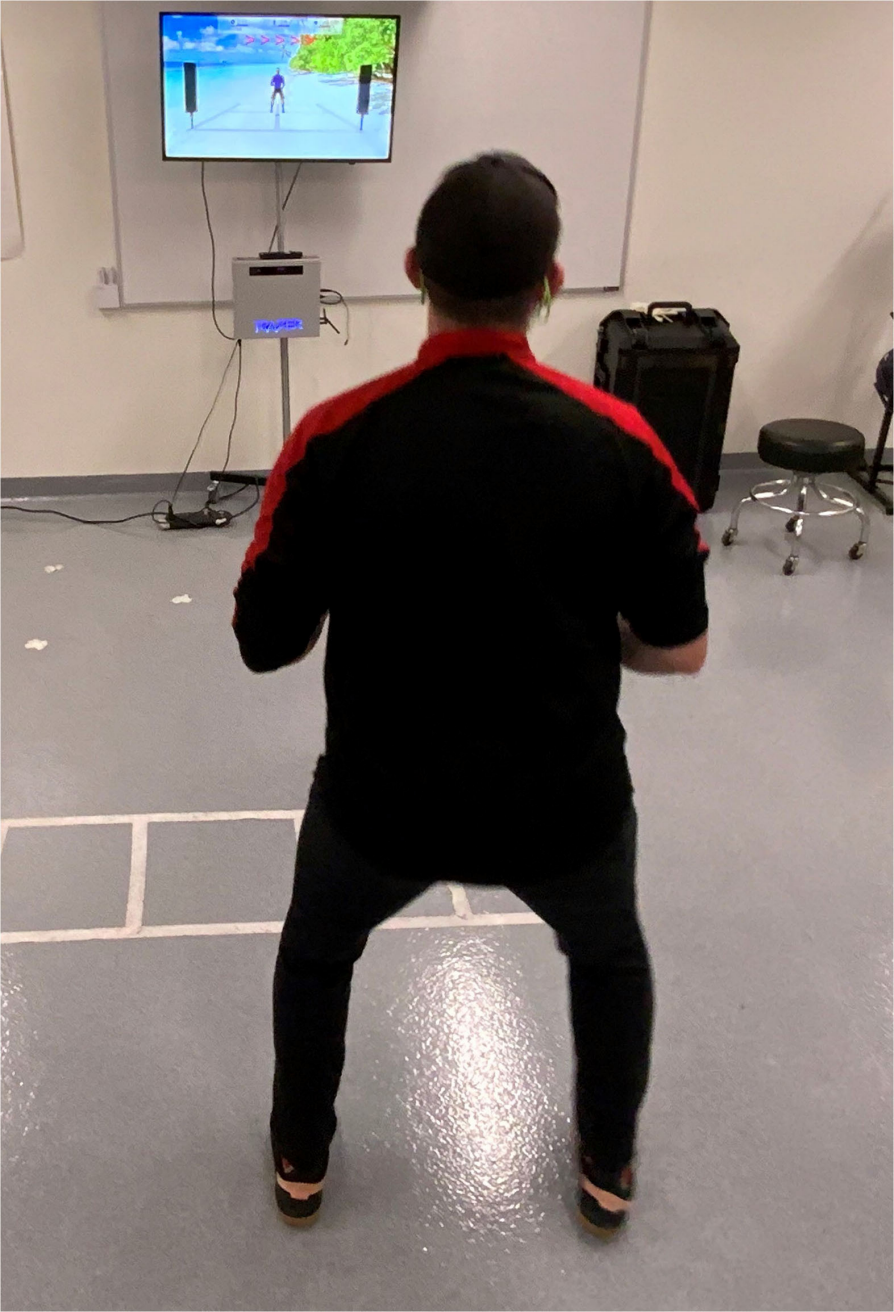
A student-athlete being assessed for their WBRA metrics.

Each player was assessed for WBRA utilizing two lateral movement tests and two diagonal-backward movement tests (TRAZER^®^ Sports Stimulator; Traq Global Ltd, Westlake, OH). The TRAZER^®^ has been found to be reliable and valid for assessing WBRA metrics (Hogg et al., 2021). The four tests used were a standard screening protocol within the unit. The order of the four tests were set by the manufacturer and were the same for each player. The player stood 2.7 m from a 48 × 86 cm television screen in the middle of an area ~1.75 × 1.75 m (Hogg et al., 2021; Figure 1). An avatar of the player was projected onto the screen and they would respond to virtual targets (Figure 2). For the lateral agility and diagonal agility tests, the athlete would respond to eight randomly appearing targets (four on each side), side-shuffling 1.8 meters to the right or left for Lateral Agility or moving backward and laterally at a 45° angle 2.7 meters for the Diagonal Agility test (Figures 1, 2). Both Lateral Agility and Diagonal Agility are defined as single-task activities. The other two tests were dual-task activities incorporating the Flanker Test, a reliable measure of executive function (Paap and Sawi, 2016). The Flanker Test is a discrimination RT type test, involving a series of five arrows appearing at the top of the screen for 500 ms, and are in one of four configurations: congruent < < < < < or > > > > >; or incongruent < < > < < or > > < > > (Eriksen and Eriksen, 1974; Paap and Sawi, 2016; Wilkerson et al., 2021b). The player was instructed to go to the target that corresponded in the direction of the middle arrow (Lateral Flanker, Diagonal Flanker). Because the athlete must first find the middle arrow, interpret the direction and then move to the corresponding target defines this as a dual-task activity. There were eight randomly appearing patterns, four repetitions each to the right and left, with two of each of the congruent patterns and two of each of the incongruent patterns. Body movement of 1.8 meters was necessary to deactivate the lateral targets, or 2.5 meters of movement to deactivate the diagonal targets (Wilkerson et al., 2018, 2020). No practice trials were permitted, but the players were allowed to watch their teammates perform the tests. The test session took each player < 6 min to complete all four tests.

Reaction time was determined by “the time elapsed from target appearance to 0.2 m of body core displacement” in the correct direction (Wilkerson et al., 2020). These times were averaged over the eight trials, four in each direction, to provide an average RT. Additionally, speed, acceleration, and deceleration () were assessed simultaneously during the same movement patterns and averages for each of these metrics were also provided. For the Flanker tests, the proportion of correct responses out of the eight trials were also produced (Wilkerson et al., 2020).

### Injury Tracking

The athletic training staffs at the schools used in this study were permitted to utilize their usual and customary procedures for injury tracking and record keeping. At the conclusion of the season a record review of the injury tracking software was performed. An injury was operationally defined as any sprains or strains suffered as a result of intercollegiate athletic participation, were evaluated by a certified athletic trainer or a physician, required a change in participant level (< 100%), for at least 1 day and were recorded on a coaches' report or daily injury report. To focus our analysis we included only those injuries resulting from insufficient neuromuscular response to dynamic loads; therefore, we excluded concussions, contusions, fractures, open wounds, and overuse disorders from the study.

### Statistical Analysis

The record review included which players had suffered an injury, how many injuries they suffered throughout the season, and the region of the body injured. Regions included: 1. core (trunk and spine), 2. lower extremity, 3. upper extremity, 4. concussion. Core and lower extremity injuries had the greatest frequency of injuries combined, but not separately, thus we chose core and lower extremity injuries (CLEI) as our dependent variable. Additionally, a review of the athletic trainers' daily injury reports provided to the coaches was performed to ensure all injuries had been accounted for and recorded. Any discrepancies or questions regarding a player's injury were taken to the athletic trainer in charge of football for consultation.

Each of the four test modes (Lateral Agility, Lateral Flanker, Diagonal Agility, and Diagonal Flanker), generated data for four metrics (RT, Speed, Acceleration, and Deceleration) creating 16 variables. Imputation for the missing values was accomplished using the group mean for the variable. There were only six scores that needed to be imputed [6**/**(16 variables × 177 players) = 2,832 total variables = 0.212%]. To examine for outliers, the raw values of the 16 variables were transformed into z-scores. Because a smaller value for RT corresponds to superior performance, the RT z-scores were multiplied by −1 to represent better performance with a higher z-score value. A review of the data was performed searching for outliers, which were operationally defined as any z-score greater than three standard deviations above or below the mean. In such cases, the outlier raw scores were replaced with a value corresponding to three standard deviations above or below the variable-specific mean value. Only 25 scores [25**/**(16 × 177 = 2,832) = 0.883%], were determined to be outliers (Tabachnick et al., 2007).

Asymmetry (Asym) was defined as the absolute difference between test metric values for the two directions to the better of the two performance values, yielding a proportion of the difference for each of the four test's metrics (RT Asym, Speed Asym, Acceleration Asym, and Deceleration Asym), producing an additional 16 variables (Wilkerson et al., 2020). The average of the four asymmetry scores for each of the four test modes was calculated (Asym Avg). An additional metric titled “Efficiency Index” was calculated by dividing the Avg RT by the Correct Proportion of Flanker Test responses. The smaller the Efficiency Index, the better the score. The mean scores and the asymmetries for each of the four metrics for each of the four test modes produced a total of 32 variables. The four Avg Asym plus both EI scores produced an additional six variables for a total of 38 variables to analyze (Halliday et al., 2018).

Multicollinearity analysis was performed to eliminate any variables that were highly correlated with other variables. Multicollinearity is present when the variance inflation factor (VIF) is >10.0 (Mertler and Vannetta, 2005; Field, 2009). Variables with high VIF values were eliminated from further consideration.

Receiver Operator Characteristic (ROC) analyses were used to assess each of the remaining variables. Our goal was to determine the best possible set of predictors and not to screen variables for statistical significance; therefore we operationally determined an ROC curve with an Area under the Curve (AUC) of ≥0.550 or a *p*-value of ≤0.20 for continuous variables, to advance to multivariable analysis (Kuijpers et al., 2006; Teyhen et al., 2007; Alba et al., 2017). The remaining continuous variables were entered into a backward logistic regression analysis to determine the best set of predictors. Predicted probabilities were calculated by SPSS for each player in the analysis for both the continuous and binary versions of the predictor variables where the natural log odds values for each participant were converted to predicted probabilities. Another ROC analysis was performed to assess the model for the probability of a CLEI occurring using the model. Further analysis of the prediction model was made utilizing the Area Under the Curve. Variables which the logistic regression identified as having the strongest association with CLEI were converted to binary variables on the basis of Youden's Index from the univariable ROC analyses (Youden's Index is the difference between the sensitivity and 1-specificity). The largest difference provided the cut-point for binary classification of the predictor variables (Youden, 1950; Teyhen et al., 2020). The logistic regression was repeated for the binary form of the surviving variables with predicted probabilities also calculated through SPSS. Receiver Operator Characteristic analysis was performed on this set of predicted probabilities and a cut-point was established using Youden's Index from this analysis (Teyhen et al., 2020).

Based on the binary variables, participants were coded with a “1” if associated with a CLEI history or a “0” if not associated with a CLEI. The coded variables were then summed, and a ROC analysis was repeated to determine the optimal number of predictors. Participants' whose summed total of positive predictor variables was at or above this cut point were coded with a “1,” those with a sum below the identified cut point were coded with a “0.” Two-by-two cross-tabulation analysis was used to calculate Sensitivity, Specificity, Positive Predictive Value, Negative Predictive Value, Odds Ratio, and Relative Risk, with associated 95% confident intervals (CI) for the individual predictors and combinations of predictors.

## Results

There were no statistical differences between the two football teams on demographic or anthropometric metrics (Table 1). The overall injury rate of 32.2% (57/177), was less than the four-season average of 45% (Luedke et al., 2020). A statistically significant difference was present in the number of injuries suffered by the two teams. The Football Bowl Subdivision team suffered only 16 injuries (16/102 = 15.7%), while the Football Championship Subdivision team had 41 injuries (41/75 = 54.7%), [${\text{χ}}_{\left(1\right)}^{2}$ = 30.08, *p* = 0.001]. Statistical power was calculated using OpenEpi website and was computed to be 99.6% (Soe et al., 2005).

**Table 1:** Demographic data for each of the football teams in the cohort.

		**Age (yrs)(±sd)**	**Height (cm) (±sd)**	**Weight (kg)(±sd)**	**BMI (±sd)**	**MMOI(±sd)**
FBS	Team A (*n* = 102)	20.07 ± 1.59	185.93 ± 0.068	105.01 ± 22.02	30.22 ± 5.32	366.77 ± 95.43
FCS	Team B (*n* = 75)	20.11 ± 1.52	185.43 ± 0.069	102.85 ± 19.53	29.79 ± 4.60	357.04 ± 86.90
	Overall (*n* = 183)	20.09 ± 1.56	1.86 ± 0.068	104.13 ± 21.00	30.05 ± 5.03	362.78 ± 91.91
	*t*-test;	*t* = −0.139	*t* = 0.480	*t* = 0.683	*t* = 0.574	*t* = 0.704
	*p*-value	*p* = 0.890	*p* = 0.632	*p* = 0.486	*p* = 0.566	*p* = 0.483

*FBS, Football Bowl Subdivision; FCS, Football Championship Subdivision; MMOI, Mass Moment of Inertia*.

Multicollinearity analysis eliminated the Avg Asym variable for each of the four tests modes. Receiver Operator Characteristic analysis was performed on the four Avg Asym variables to double check for prediction power, but these AUCs were <0.550. Receiver Operator Characteristic analyses were performed on the remaining 34 variables and further reducing the data set to five variables. The AUCs for these five variables ranged from 0.555 to 0.596 and *p*-values from 0.040 to 0.242.

The five continuous independent variables that survived ROC analysis were entered into a backward entry logistic regression. This regression analysis was performed to ascertain the effects of RT, Speed, Acceleration, and Deceleration assessed through the four different test modes (Lateral Agility, Lateral Flanker, Diagonal Agility, and Diagonal Flanker), on the likelihood for subsequent CLEI occurrence. There were three variables which survived this step: Lateral Flanker Deceleration Asym, Lateral Agility Acceleration Asym, and Diagonal Agility RT Avg. A comparison of the means and standard deviations of these final three predictor variable for those classified as injured vs. not injured is provided in Table 2. An independent *t*-test comparing the Injured vs. Not Injured groups was statistically significant for the Lateral Flanker Deceleration Asym (*p* = 0.034), and Lateral Agility Acceleration Asym (*p* = 0.030), predictors, but there was not a statistically significant difference between the groups for the Diagonal Agility RT Avg (*p* = 0.127).

**Table 2:** Means and standard deviations comparing injured vs. not injured for the final three predictor variables.

	**Injured**	**Not injured**	**Independent** ***t*****-test (***p*** ≤ 0.05)**
*n* =	57	120	177
LF Dec Asym	14.29% ± 9.45	11.25% ± 8.52	*t*_(175)_ = −2.14; *p* = 0.034
LA Acc Asym	13.60% ± 9.22	10.66% ± 9.22	*t*_(175)_ = −2.94; *p* = 0.030
DA RT Avg	0.761 ± 0.167	0.724 ± 0.144	*t*_(175)_ = −1.53; *p* = 0.127

*LF, Lateral Flanker; LA, Lateral Agility; DA, Diagonal Agility; Dec, Deceleration; Acc, Acceleration; RT, Reaction Time; Avg, Average*.

The three-factor model was statistically significant [${\text{χ}}_{\left(3\right)}^{2}$ = 13.41, *p* = 0.004], and correctly classified 68.9% (122/177) of the cases. The Nagelkerke *R*^2^ was 0.102 while the Hosmer and Lemeshow Test was not statistically significant (*p* = 0.340), signifying that the model demonstrated acceptable fit to the data. The beta weights from the logistic regression along with the Wald statistic, *p*-value and the adjusted odds ratio with 95% CIs are provided for the continuous variables in Table 3. One concern was that the 95% CIs for the adjusted odds ratio for the continuous version of the final three predictors were wide, suggesting unstable data.

**Table 3:** Logistic regression results for the final three, predictor, continuous variables.

**Predictor variable**	**Beta weight**	**Wald Statistic**	***p*** **-value**	**Exp (B) (95% CI)**
LF Dec Asym	4.25	5.13	0.024	70.08 (1.77, 2,773.59)
LA Acc Asym	4.78	5.79	0.016	118.83 (2.42, 5,831.78)
DA RT Avg	2.30	4.30	0.038	9.94 (1.14, 86.82)

*Degrees of Freedom, 1*.

*LF, Lateral Flanker; LA, Lateral Agility; DA, Diagonal Agility; Dec, Deceleration; Acc, Acceleration; RT, Reaction Time; Avg, Average; Adj OR, Adjusted Odds Ratio*.

Based on the univariable ROC analysis for each of the final three predictors, cut-points were determined based on Youden's Index. The logistic regression was repeated for the binary form of the final three predictor variables. This binary, three-factor model was statistically significant [${\text{χ}}_{\left(3\right)}^{2}$ = 20.75, *p* ≤ 0.001], and it correctly classified 68.9% (122/177) of the cases. The Nagelkerke *R*^2^ was 0.156 while the Hosmer and Lemeshow Test was not statistically significant (*p* = 0.607), signifying that the model demonstrated acceptable fit to the data. The beta weights from the logistic regression along with the Wald statistic, *p*-value and the adjusted odds ratio with 95% CIs are provided for the binary variables in Table 4. Receiver Operator Characteristic analysis was performed on the predicted probabilities for the binary form of the final three predictors and found an AUC = 0.709 (*p* ≤ 0.001). (Sensitivity = 63.2%; Specificity = 76.3%).

**Table 4:** Logistic regression results for the final three, predictor, binary variables.

**Predictor variable**	**Beta weight**	**Wald statistic**	***p*** **-value**	**Adj OR (95% CI)**
LF Dec Asym	1.07	8.34	0.004	2.90 (1.41, 5.98)
LA Acc Asym	1.01	7.36	0.007	2.74 (1.32, 5.66)
DA RT Avg	0.896	6.17	0.013	2.45 (1.21, 4.97)

*Degrees of Freedom, 1*.

*LF, Lateral Flanker; LA, Lateral Agility; DA, Diagonal Agility; Dec, Deceleration; Acc, Acceleration; RT, Reaction Time; Avg, Average; Adj OR, Adjusted Odds Ratio*.

Univariable analysis of the binary form of the final three predictors was performed with a 2 x 2 cross-tabulations table (Table 5). The odd ratios for each of the three variables ranged from 1.98 to 2.75, while the Positive Predictive Value ranged from 41.2 to 47% and Negative Predictive Value ranged from 73 to 79.7%.

**Table 5:** 2 × 2 cross-tabulations output for final three binary variables in the prediction model.

**Variable**	**Cut-point**	**AUC**	**Accuracy**	**Sn (95% CI)**	**Sp (95% CI)**	**PPV (95% CI)**
LF Dec Asym	≥8.1%	0.596	57.4%	73.7% (61.0%, 83.4%)	49.6% (40.8%, 58.4%)	41.2% (35.6%, 47.0%)
LA Acc Asym	≥14.7%	0.592	65.9%	40.4% (28.6%, 53.3%)	78.2% (69.9%, 84.6%)	46.9% (35.8%, 58.5%)
DA RT Avg s	≥0.787 sec	0.565	62.7%	43.9% (31.8%, 56.7%)	71.7% (63.0%, 79.0%)	42.4% (32.8%, 52.5%)
**Variable**	**NPV (95% CI)**	**OR (95% CI)**	**RR (95% CI)**	**Chi-square**	***p*** **-value**	**Fisher's exact** ***p*****-value**
LF Dec Asym	79.7% (71.1%, 86.3%)	2.75 (1.38, 5.49)	2.03 (1.66, 2.50)	8.55	0.003	0.003
LA Acc Asym	73.2% (68.4%, 77.6%)	2.42 (1.22, 4.80)	1.75 (1.43, 2.15)	6.57	0.010	0.009
DA RT Avg	72.9% (67.6%, 77.6%)	1.98 (1.03, 3.81)	1.56 (1.27, 1.92)	4.19	0.041	0.031

*LF, Lateral Flanker; LA, Lateral Agility; DA, Diagonal Agility; Dec, Deceleration; Acc, Acceleration; RT, Reaction Time; Asym, Asymmetry; Avg, Average; AUC, Area Under the Curve; Sn, Sensitivity; CI, Confidence Interval; Sp, Specificity; PPV, Positive Predictive Value; NPV, Negative Predictive Value; OR, Odds Ratio; RR, Relative Risk*.

**Figure 3:**
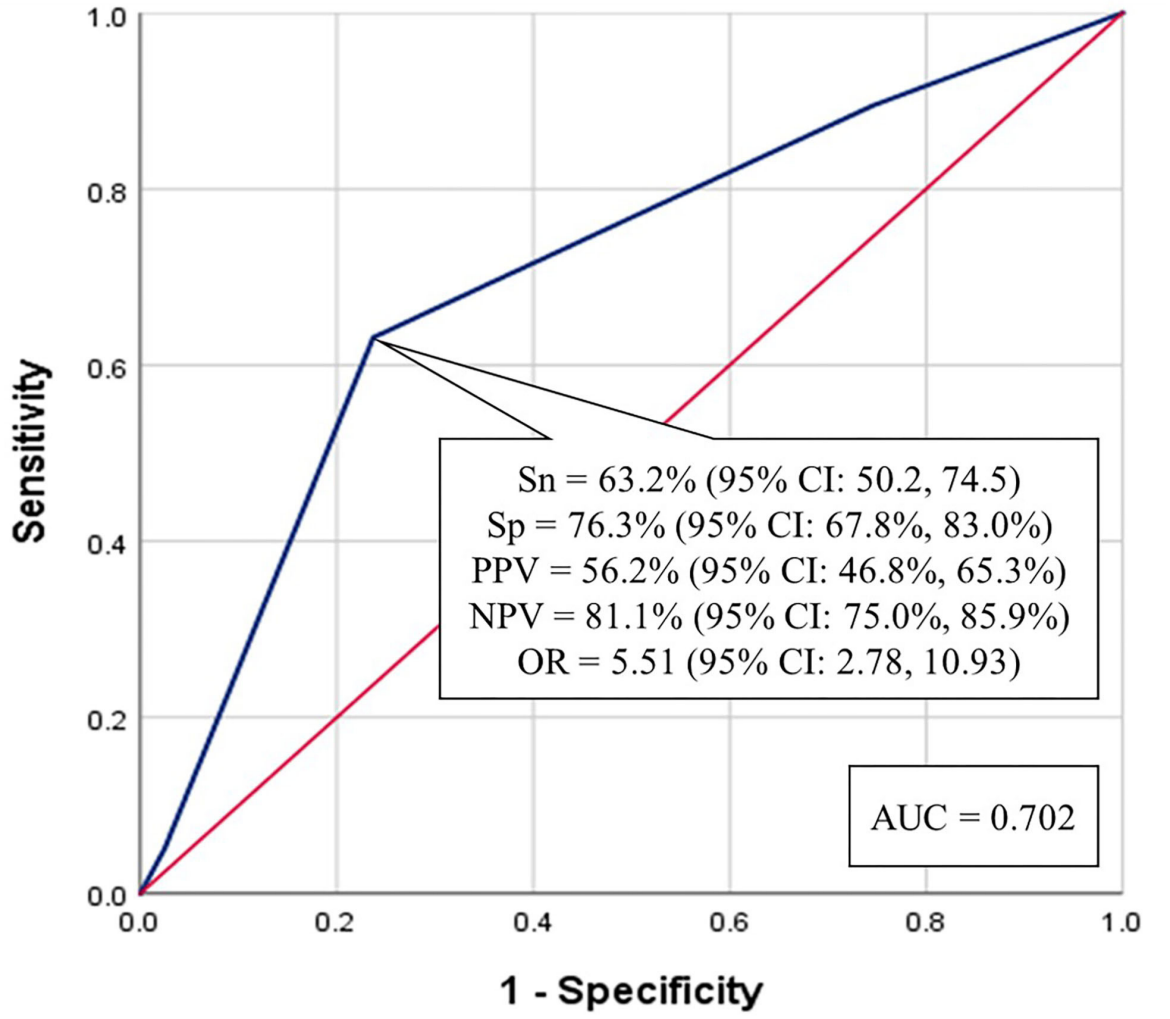
Receiver operator characteristic analysis for the optimum number of predictors (≥2) from the three-factor model.

The number of predictors each player was positive for was summed. A ROC analysis was performed on the three-factor model to determine the optimum cut-point, which was found to be ≥2 factors (Figure 3). The AUC for this model was 0.702. To examine the multiple metrics of the three-factor model a 2 × 2 cross-tabulations table was created (Table 6). A football player who was positive on any combination of two of the three variables had an OR was 5.51, indicating he had just over 5.5 times greater odds of suffering a CLEI compared to another player who was positive on less than two factors. The Sensitive was 63.2%, Specificity was 76.3%, regarding model accuracy. The Positive Predictive Value of a football player suffering a CLEI was 56.3% (36/64) and Negative Predictive Value was 81.1% (90/111).

**Table 6:** 2 × 2 cross-tabulation table for the 3-factor model, ≥2 factors.

	LA Acc Asym ≥ 40.4%	
	LF Dec Asym ≥ 73.7%	
	DA RT Avg ≥ 0.439 s	
	**CLEI**	**No CLEI**
≥2 Factors	36	28
<2 Factors	21	90
Fisher's exact test (one-sided) *p* < 0.001		
Sn = 63.2% (95% CI: 50.2%, 74.5%)		Sp = 76.3% (95% CI: 67.8%, 83.0%)
PPV = 56.2% (95% CI: 46.8%, 65.3%)		NPV = 81.1% (95% CI: 75.0%, 85.9%)
OR = 5.51 (95% CI: 2.78, 10.93)		Accuracy = 72.0% (126/175)

*LF, Lateral Flanker; LA, Lateral Agility; DA, Diagonal Agility; Acc, Acceleration; Dec, Deceleration; RT, Reaction Time; Asym, Asymmetry; Avg, Average; Sn, Sensitivity; Sp, Specificity; PPV, Positive Predictive Value; NPV, Negative Predictive Value; OR, Odds Ratio*.

### Predicted Probabilities

**Figure 4:**
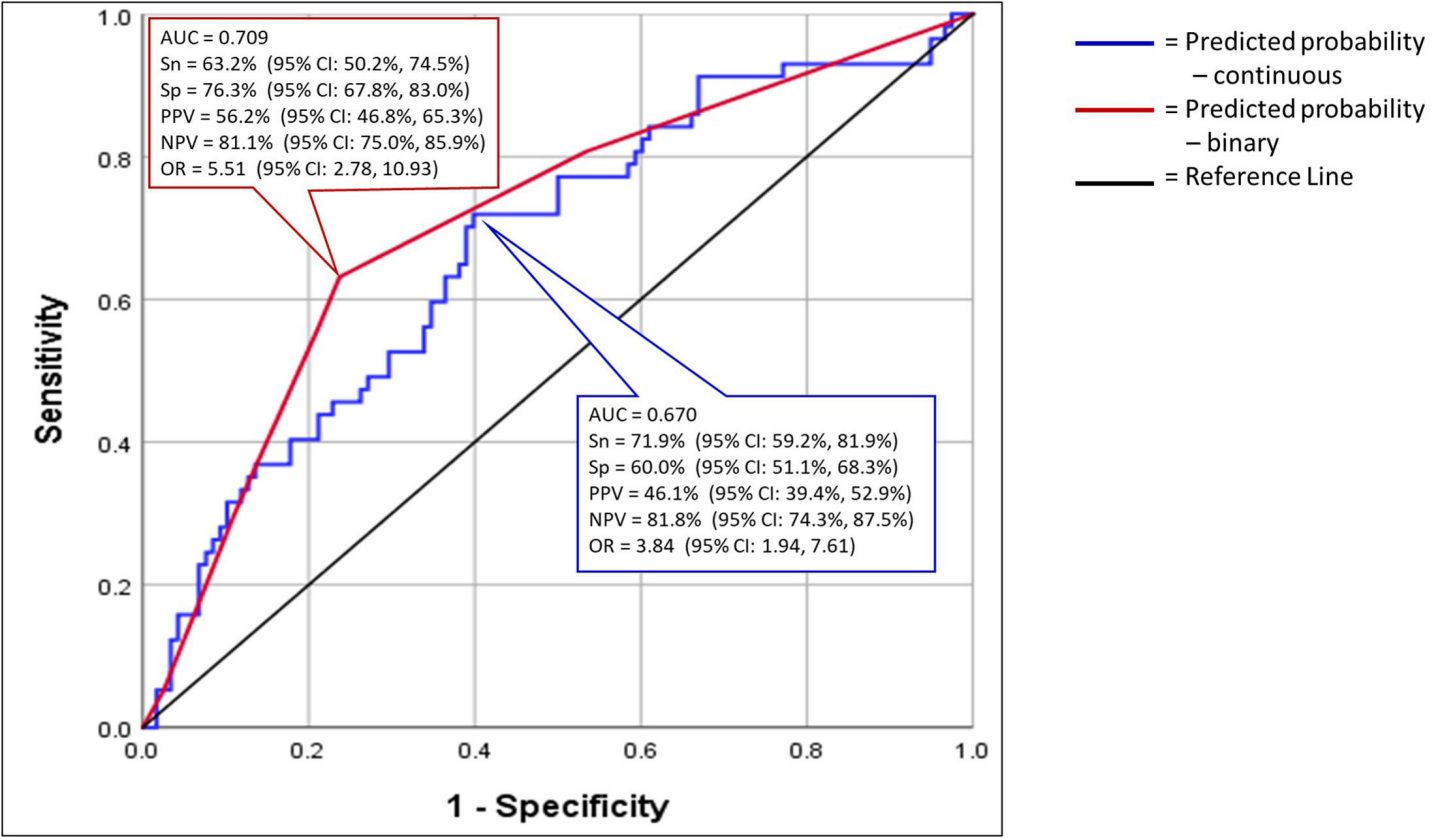
Receiver operator characteristic analysis for the predicted probabilities of the final three variables for both the continuous and binary forms of the three predictors.

The predicted probabilities were calculated as part of the logistic regression analysis for the continuous variables. A ROC analysis of the predicted probabilities for the continuous variables was produced and had an AUC of 0.670 (*p* ≤ 0.001) (Figure 4). The prognostic accuracy was determined utilizing Youden's Index (Youden, 1950). The Youden's Index cut-point provided 71.9% Sensitivity, 60.0% Specificity, and an Odd Ratio of 3.84 (95% CI: 1.94, 7.61). The Positive Predictive Value of a football player suffering a CLEI was 46.1% and Negative Predictive Value was 81.8% (Figure 4).

The logistic regression analysis was repeated with the binary form of the final three variables and the predicted probabilities were calculated. A ROC analysis of the predicted probabilities for the binary variables was produced and had an AUC of 0.709 (*p* > 0.001) (Figure 4). The prognostic accuracy was determined using the Youden's Index (Youden, 1950). The Youden's Index cut-point provided 63.2% Sensitivity, 76.3% Specificity, Odd Ratio of 5.38 (95% CI: 2.72, 10.63). The Positive Predictive Value of a football player suffering a CLEI was 56.2% and Negative Predictive Value was 81.1% (Figure 4; Table 6).

### Injury Incidence

Table 7 provides the injury incidence by the number of risk-factors a player possessed. Players with ≥2 factors suffered the majority of the injuries with 55.4% of those players with two or more predictors becoming injured. Only 18.8% of the players with <2 factors suffered a CLEI.

**Table 7:** Injury incidence relative to number of risk factors.

**Number of factors**	**CLEI**	**No CLEI**	**Total**	**Incidence**	**Cumulative incidence**
0	6	31	37	16.2%	21/112 = 18.8%
1	15	60	75	20.0%	
2	33	26	59	55.9%	36/65 = 55.4%
3	3	3	6	50.0%	
Total	57	120	177	32.2%	

*CLEI, Core and Lower Extremity Injury*.

## Discussion

This study responded to a need of which WBRA metrics could identify the Division I football players who were at elevated risk for CLEI. Additionally, we demonstrated methods to assess RT, Speed, Acceleration, and Deceleration simultaneously. The results demonstrated that RT, Acceleration, and Deceleration were the key metrics for this cohort of Division I football players. Finally, a high asymmetry value, the difference between right and left movements, maybe an important injury risk factor.

### Prediction Model

Our intent was to identify the WBRA assessment metrics that help to identify those Division I football players might be classified as high-risk for CLEI. Initially, we used ROC analyses to determine the individual strength of the predictor variable using a liberal threshold for the AUC of ≥0.550 or a *p*-value of ≤ 0.20 for variables to advance to multivariable analysis (Kuijpers et al., 2006; Teyhen et al., 2007; Alba et al., 2017). We decided that these criteria would prevent us from excluding potentially important predictors. There were five predictors that were retained by this process and we created our model using logistic regression with the continuous representations of these variables. The logistic regression analysis retained three predictors (Lateral Agility Acceleration Asym, Lateral Flanker Deceleration Asym, and Diagonal Agility RT Avg), as the strongest set. To assess the potential for a simplification of the prediction model, univariable analyses were performed to create binary cut-points. Binary logistic regression is a commonly used method to create multivariable clinical prediction models (Hosmer and Lemeshow, 2000; Moons et al., 2015). The “events per variable” (EPV) is the ratio of events (i.e., CLEI occurrence), to the number of predictor variables, which has been recommended to be no <10:1 (Moons et al., 2014; Pavlou et al., 2016; Austin and Steyerberg, 2017). For our final model, we had a ratio of 19 injuries per predictor variable (57 injuries/3 predictor variables).

We also acknowledge a common criticism of binary prediction models in that some participants may be misclassified because they are just above or below the established cut-point. It is understood the cut-points we established may not be exactly the same cut-points for similar samples; however, we are confident that our predictor variables would be similar to a similar sample. We provided the reader with the accuracy for the predictors for our model and for the combination of predictors with 2 × 2 cross-tabulation. The accuracy of the individual predictors ranged from 57.4 to 65.9% (Table 5), and was 72.0% for players having ≥ 2 factors (Table 6). We further understand the potential for a non-linear relationship between the predictors and CLEI. Further study is warranted to account for a possible non-linear relationship, thus potentially strengthening our prediction model.

A common criticism of prediction modeling is the lack of adequate sample size. Our sample of 177 football players was found to produce a statistical power level of 99.6% for avoidance of a Type II error (Soe et al., 2005). Stratification to explore interaction effects could potentially lower the cell counts to single digits (Prieto-Marañón et al., 2012).

A key problem with many prediction models is lack of external validation. Future research should determine the generalizability of our model to a validation cohort. Large multi-site data collection may lead to model refinement that will provide strong external validity.

The ROC curve, specifically the AUC, relates to classification accuracy for this cohort. For the predicted probabilities for the continuous variables the ROC curve had an AUC of 0.670 (95% CI: 0.584, 0.755). For the predicted probabilities for the binary version of the variables, the ROC curve was even stronger with an AUC of 0.709 (95% CI: 0.625, 0.792). According to Alba et al. (2017), ROC curve AUC of 0.60–0.75 are interpreted as “possibly helpful discrimination.”

Clinically, when examining prediction models, examining the predicted probabilities might be a logical way to compare not only results across groups, but it also provides each individual with a probability of his or her likelihood of sustaining the specified outcome event.

### Reaction Time

We operationally defined RT has the time between the appearance of the target until the body's core moved 0.2 m in the correct direction (Wilkerson et al., 2020). Several studies have demonstrated the importance of reaction time to athletic success (Caccese et al., 2021; Lempke et al., 2020). Reaction time is also affected when someone suffers a concussion (Gorus et al., 2006; Caccese et al., 2021; Lempke et al., 2020; Wilkerson et al., 2020). We found Diagonal Agility RT Avg to be predictor of CLEI. Dual-task processing tests such as the Lateral Flanker, would have slowed RTs from having to process the arrow sequence and decide the direction in which to move to the proper target.

### Asymmetry

Optimal movement is the ability to perform a functional task equally well to either side as the task demands. Asymmetry between extremities or movement directions may be present following musculoskeletal injury, surgery, or a concussion, which may constrain an athlete's movement repertoire during participation in demanding activities (Hughes et al., 2020; Wilkerson et al., 2020). A deficiency in one's ability to move in either direction equally well can potentially elevate injury risk. Several studies have demonstrated asymmetry as a risk factor in injury prediction (Chalmers et al., 2017; Eagle et al., 2019; Hughes et al., 2020; Wilkerson et al., 2020; King et al., 2021). Although not part of the current study, others have speculated an imbalance in the distribution of excitatory and inhibitory moderators between the hemispheres of the brain may be a contributing factor in movement asymmetries. One's ability to adjust and accommodate to ever changing environments is often the difference between success and failure (Serrien et al., 2006; Takeuchi et al., 2012; Garrett et al., 2013; Grady and Garrett, 2018; Wilkerson et al., 2020). If not adequately addressed, asymmetries can persist long after the athlete has returned to play from an injury. However, some clinicians fail to either recognize the existence of the asymmetry or neglect to assess for the asymmetry. This can cause a clinician to believe the athlete has returned to “normal,” when in reality the asymmetry problem that was not assessed may manifest itself at a later time (Wilkerson et al., 2020, 2021a; King et al., 2021).

What has not been universally accepted is the threshold for these differences? In a study of professional soccer players, Read et al., attempted to do quantify this threshold with a variety of tests, including hopping and jumping. They concluded that they were unable to find a single asymmetry threshold, stating that it was their belief that the thresholds were sport and age specific (Read et al., 2021). Others have stated that an asymmetry of >10% to be problematic (Schiltz et al., 2009; Lambert et al., 2020). The biggest challenge is a lack of prospective studies to identify the asymmetries from a set of baseline measures. The data used for our study was gathered at baseline, prior to the start of pre-season practice, when both teams were nearing the end of their pre-season strength and conditioning programs with the common goal of being in excellent physical condition for the upcoming season. Our model identified two asymmetries as predictors of CLEI: Lateral Agility Acceleration Asym (≥14.7%), and Lateral Flanker Deceleration Asym (≥8.1%). The univariable ORs for these predictors of CLEI were 2.75 (95% CI: 1.38, 5.49), and 2.42 (95% CI: 1.22, 4.80), respectively. Although these cut-points may not provide equivalent discrimination in similar cohorts, it does not diminish the potential importance of asymmetrical movement as a factor that may elevate CLEI risk.

### Acceleration and Deceleration

Each sport has its unique acceleration and deceleration demands. Acceleration, one's ability to speed up, has a greater metabolic cost. Deceleration, one's ability to slow down, has a greater mechanical burden, due to greater peak loads and loading rate (Lieberman et al., 2015; Harper et al., 2019, 2020). Deceleration forces include horizontal and vertical forces to include braking power and braking impulse (Lieberman et al., 2015; Harper et al., 2019, 2020), but it is the horizontal forces which are costlier than the vertical forces. The horizontal forces contribute more than a third of the total cost of running (Lieberman et al., 2015). Just like in an automobile where braking puts more stress and strain on the vehicle's systems than acceleration, braking of the body creates increased forces and cause more structural damage than acceleration (Harper et al., 2019). High-intensity accelerations and decelerations have been studied in soccer players. In a typical soccer match, a player may perform from 6 to 8 high-intensity acceleration and deceleration movement per minute of game time (Harper et al., 2020). In college football, defensive backs displayed higher acceleration and deceleration values than other position groups. As a unit, defensive players experienced great acceleration-deceleration values (Wellman et al., 2017). Our prediction model found one acceleration factor and one deceleration factor as indicators of elevated CLEI risk.

In a meta-analysis by Harper et al., they examined high and very high intensities for acceleration and deceleration. Eleven of the studies established 3.0 m/s^−2^ as the high intensity threshold while six studies established 3.5 m/s^−2^ as the very-high intensity threshold (Harper et al., 2019). We applied the Harper et al., thresholds to assess the possible relevance of their findings to our data of the Lateral Agility Acceleration Avg and the Lateral Flanker Deceleration Avg. The largest percentage of players at a particular intensity was for the high intensity threshold – Lateral Agility Acceleration Avg with 50% of the players at ≥3.0 m/s^−2^. There was about one-third or fewer of the players at or above for the other three metrics, very high intensity threshold – Lateral Agility Acceleration Avg, very high intensity threshold – Lateral Flanker Acceleration Avg, and high intensity threshold – Lateral Flanker Acceleration Avg. An examination of the ORs found all four metrics to have ORs < 2.0 and all of the 95% CIs lower limits were below the 1.0 threshold. For our data set, these thresholds do not have a meaningful impact.

### Dual-Task Processing

The ability to proficiently process a variety of neural inputs and produce a correct physical response is the essence of athletic movements (Nuzzo, 2015; Tarara et al., 2016; Araújo et al., 2019). Athletic-related movements by their nature are dual-task activities. Rarely do athletic activities occur as single-task actions. Movement happens with some degree of cognitive processing in order for the proper response to occur. Research has demonstrated those with a history of concussion or anterior cruciate ligament reconstruction have slowed dual-task performance (Marques et al., 2020; Ness et al., 2020). This slowing of dual-task neural processing may lead to differences in performance capabilities which may also lead to an elevated risk for musculoskeletal injury (Wilkerson et al., 2017, 2020). Several authors have presented evidence of a possible residual, low-level inflammatory response in the brain following concussion, which causes altered neurocognitive processing (Ezza and Khadrawyb, 2014; Churchill et al., 2017, 2019). If true, then not only would cognitive function be impaired, but so would dual-task WBRA actions. The degree to which dual-task processing is integral to functional movement makes it remarkable that clinicians do not spend more time assessing this phenomenon. Further study is warranted to examine more closely the effects of neurocognitive dysfunction and its effect on physical performance which could yield benefits for all levels of competition.

The Flanker Test adds a cognitive component to the movements as the player must first discern the middle arrow, then interpret that arrow's direction, and finally move its direction (Wilkerson et al., 2021a). As expected, the metrics for the lateral flanker test were worse than the metrics on the Lateral Agility. Surprisingly, this was not the case for the Diagonal Flanker test vs. the Diagonal Agility test as the Diagonal Flanker variables did not demonstrate substantial discriminatory power. One possible reason for this phenomenon is that the diagonal movements required for the WBRA tests mimic those that offensive lineman perform in a football game or practice, using their pass protection skills. Anecdotally, it appeared that offensive linemen and defensive backs did better on the diagonal tests than defensive linemen and wide receivers. Unfortunately, the data set did not contain specific position data of each player, only their binary classification as a lineman or a skill position player. Further study examining these specific positions groups would be beneficial to further understand the role biomechanics play in these assessments.

### Limitations

Although the order of the specific repetitions within each test mode was randomized, the order of the test modes was not randomized as the protocol used is pre-set by system software. The order in which the WBRA tests were administered was the same for each participant: Lateral Agility, Lateral Flanker, Diagonal Agility, and Diagonal Flanker. It cannot be ruled out the role of doing the agility test prior to the Flanker test may have had an impact on the Flanker exam outcome. The players were able to watch their teammates perform the test battery. It cannot be determined how a player watching his teammates perform the test prior to their turn may have had on their performance when they completed the test battery.

Another limitation to our study, was the effort given by the players. It would be expected with full effort by the players there would be a statistically significant differences between the Football Bowl Subdivision team and the Football Championship Subdivision team; however, this was not necessarily the case. Analysis of the means of test metrics found the players from the Football Championship Subdivision team outperformed the players from the Football Bowl Subdivision team on several of the test metrics. Finally, as a means to limit the time each player spent doing the test battery, only eight repetitions were performed for each of the WBRA test modes. Four repetitions to either side may not have been sufficient to ensure reliable measurements.

Caution should be exercised applying our results to other college football teams. Further examination utilizing players from other levels of intercollegiate football would be beneficial. Ideally, large multi-site data collection may lead to model refinement that will provide strong external validity.

## Data Availability Statement

The raw data supporting the conclusions of this article will be made available by the authors, without undue reservation.

## Ethics Statement

The studies involving human participants were reviewed and approved by Institutional Review Board, Arkansas State University, State University, 72467-2760. The patients/participants provided their written informed consent to participate in this study.

## Author Contributions

The manuscript draft was written by SLB, with the content of the final draft revised in response by GBW and reviewers. All components of the data analysis were done by SLB and reviewed by GBW to ensure that the results were derived from accurate data collected by SLB and GBW. All authors made significant contributions to the conceptualization, design, and implementation of the study.

## Conflict of Interest

Whole-body reactive agility data were acquired from equipment purchased by Arkansas State University and the University of Tennessee at Chattanooga from Traq Global, Ltd. (Westlake, OH). None of the authors currently has an affiliation with Traq Global, Ltd., and neither author has any conflicting interests to disclose.

## Publisher's Note

All claims expressed in this article are solely those of the authors and do not necessarily represent those of their affiliated organizations, or those of the publisher, the editors and the reviewers. Any product that may be evaluated in this article, or claim that may be made by its manufacturer, is not guaranteed or endorsed by the publisher.

## References

[B1] Alba A. C. Agoritsas T. Walsh M. Hanna S. Iorio A. Devereaux P.. (2017). Discrimination and calibration of clinical prediction models: users' guides to the medical literature. J. Am. Med. Assoc. 318, 1377–1384. 10.1001/jama.2017.12126 29049590

[B2] Araújo D. Hristovski R. Seifert L. Carvalho J. Davids K. (2019). Ecological cognition: expert decision-making behaviour in sport. Int. Rev. Sport Exerc. Psychol. 12, 1–25. 10.1080/1750984X.2017.1349826

[B3] Austin P. C. Steyerberg E. W. (2017). Events per variable (EPV) and the relative performance of different strategies for estimating the out-of-sample validity of logistic regression models. Stat. Meth. Med. Res. 26, 796–808. 10.1177/0962280214558972 25411322 PMC5394463

[B4] Bagherian S. Ghasempoor K. Rahnama N. Wikstrom E. A. (2019). The effect of core stability training on functional movement patterns in college athletes. J. Sport Rehabil. 28, 444–449. 10.1123/jsr.2017-0107 29405798

[B5] Bahr R. (2016). Why screening tests to predict injury do not work—and probably never will: a critical review. Br. J. Sports Med. 50, 776–780. 10.1136/bjsports-2016-096256 27095747

[B6] Beltran-Alacreu H. Lopez-de-Uralde-Villanueva I. Calvo-Lobo C. La Touche R. Cano-de-la-Cuerda R. Gil-Martínez A.. (2018). Prediction models of health-related quality of life in different neck pain conditions: a cross-sectional study. Patient Prefer. Adher. 12:657. 10.2147/PPA.S162702 29750020 PMC5936011

[B7] Bruce J. M. Echemendia R. J. Meeuwisse W. Hutchison M. G. Aubry M. Comper P. (2018). Development of a risk prediction model among professional hockey players with visible signs of concussion. Br. J. Sports Med. 52, 1143–1148. 10.1136/bjsports-2016-097091 28377444

[B8] Bruce S. L. Rush J. R. Torres M. M. Lipscomb K. J. (2017). Test-retest and interrater reliability of core muscular endurance tests used for injury risk screening. Int. J. Athl. Ther. Train. 22, 14–20. 10.1123/ijatt.2016-0001

[B9] Büttner F. Howell D. R. Ardern C. L. Doherty C. Blake C. Ryan J.. (2020). Concussed athletes walk slower than non-concussed athletes during cognitive-motor dual-task assessments but not during single-task assessments 2 months after sports concussion: a systematic review and meta-analysis using individual participant data. Brit. J. Sport. Med. 54, 94–101. 10.1136/bjsports-2018-100164 31331944

[B10] Caccese J. B. Eckner J. T. Franco-MacKendrick L. Hazzard J. B. Ni M. Broglio S. P.. (2021). Interpreting clinical reaction time change and recovery after concussion: a baseline versus norm-based cutoff score comparison. J Athl Train. 56, 851–859. 10.4085/JAT0457-20 34375406 PMC8359707

[B11] Chalmers S. Fuller J. T. Debenedictis T. A. Townsley S. Lynagh M. Gleeson C.. (2017). Asymmetry during preseason functional movement screen testing is associated with injury during a junior Australian football season. J. Sci. Med. Sport. 20, 653–657. 10.1016/j.jsams.2016.12.076 28233674

[B12] Churchill N. W. Hutchison M. G. Graham S. J. Schweizer T. A. (2019). Mapping brain recovery after concussion: from acute injury to 1 year after medical clearance. Neurology 93:e1980–e92. 10.1212/WNL.0000000000008523 31619480 PMC6885578

[B13] Churchill N. W. Hutchison M. G. Richards D. Leung G. Graham S. J. Schweizer T. A. (2017). Neuroimaging of sport concussion: persistent alterations in brain structure and function at medical clearance. Sci. Rep. 7, 1–9. 10.1038/s41598-017-07742-3 28839132 PMC5571165

[B14] De Blaiser C. Roosen P. Willems T. Danneels L. Bossche L. V. De Ridder R. (2018). Is core stability a risk factor for lower extremity injuries in an athletic population? A systematic review. Phys. Ther. Sport. 30, 48–56. 10.1016/j.ptsp.2017.08.076 29246794

[B15] Eagle S. R. Kessels M. Johnson C. D. Nijst B. Lovalekar M. Krajewski K.. (2019). Bilateral strength asymmetries and unilateral strength imbalance: predicting ankle injury when considered with higher body mass in US special forces. J. Athl. Train. 54, 497–504. 10.4085/1062-6050-255-18 31074634 PMC6602377

[B16] Eriksen B. A. Eriksen C. W. (1974). Effects of noise letters upon the identification of a target letter in a non-search task. Percept. Psychophys. 16, 143–149. 10.3758/BF03203267

[B17] Ezza H. Khadrawyb Y. (2014). Glutamate excitotoxicity and neurodegeneration. J. Mol. Genet. Med. 8:141. 10.4172/1747-0862.1000141

[B18] Field A. (2009). Discovering statistics using SPSS, in Wright D. B. ed. 3rd Edn. (Thousasnd Oaks, CA: SAGE Publications).

[B19] Fonseca S. T. Souza T. R. Verhagen E. Van Emmerik R. Bittencourt N. F. Mendonça L. D.. (2020). Sports injury forecasting and complexity: a synergetic approach. Sports Med. 50, 1757–1770. 10.1007/s40279-020-01326-4 32757162

[B20] Frangiamore S. Dornan G. J. Horan M. P. Mannava S. Fritz E. M. Hussain Z. B.. (2020). Predictive modeling to determine functional outcomes after arthroscopic rotator cuff repair. Am. J. Sports Med. 48, 1559–1567. 10.1177/0363546520914632 32406765

[B21] Gallagher V. T. Murthy P. Stocks J. Vesci B. Colegrove D. Mjaanes J.. (2020). Differential change in oculomotor performance among female collegiate soccer players versus non-contact athletes from pre-to post-season. Neurotrauma Rep. 1, 169–180. 10.1089/neur.2020.0051 33274345 PMC7703496

[B22] Garrett D. D. Samanez-Larkin G. R. MacDonald S. W. Lindenberger U. McIntosh A. R. Grady C. L. (2013). Moment-to-moment brain signal variability: a next frontier in human brain mapping? Neurosci. Biobehav. Rev. 37, 610–624. 10.1016/j.neubiorev.2013.02.015 23458776 PMC3732213

[B23] Giza C. C. Hovda D. A. (2014). The new neurometabolic cascade of concussion. Neurosurgery 75:S24–S33. 10.1227/NEU.0000000000000505 25232881 PMC4479139

[B24] Gordon D. Hayward S. van Lopik K. Philpott L. West A. (2021). Reliability of bilateral and shear components in a two-legged counter-movement jump. Proc. Ins. Mech. Eng. B J. Eng. 10.1177/1754337121995967

[B25] Gorus E. De Raedt R. Mets T. (2006). Diversity, dispersion and inconsistency of reaction time measures: effects of age and task complexity. Aging Clin. Exp. Res. 18, 407–417. 10.1007/BF03324837 17167305

[B26] Grady C. L. Garrett D. D. (2018). Brain signal variability is modulated as a function of internal and external demand in younger and older adults. Neuroimage 169, 510–523. 10.1016/j.neuroimage.2017.12.031 29253658

[B27] Gribble P. A. Terada M. Beard M. Q. Kosik K. B. Lepley A. S. McCann R. S.. (2016). Prediction of lateral ankle sprains in football players based on clinical tests and body mass index. Am. J. Sports Med. 44, 460–467. 10.1177/0363546515614585 26646517

[B28] Halliday D. W. Stawski R. S. Cerino E. S. DeCarlo C. A. Grewal K. MacDonald S. W. (2018). Intraindividual variability across neuropsychological tests: dispersion and disengaged lifestyle increase risk for Alzheimer's disease. J. Intell. 6:12. 10.3390/jintelligence6010012 31162439 PMC6480779

[B29] Hammill R. R. Beazell J. R. Hart J. M. (2008). Neuromuscular consequences of low back pain and core dysfunction. Clin. Sports Med. 27, 449–462. 10.1016/j.csm.2008.02.005 18503877

[B30] Harper D. J. Carling C. Kiely J. (2019). High-intensity acceleration and deceleration demands in elite team sports competitive match play: a systematic review and meta-analysis of observational studies. Sports Med. 49, 1923–1947. 10.1007/s40279-019-01170-1 31506901 PMC6851047

[B31] Harper D. J. Morin J.-B. Carling C. Kiely J. (2020). Measuring maximal horizontal deceleration ability using radar technology: reliability and sensitivity of kinematic and kinetic variables. Sports Biomech. 1–17. 10.1080/14763141.2020.1792968 [Epub ahead of print]. 32731845

[B32] Hart J. M. Fritz J. M. Kerrigan D. C. Saliba E. N. Gansneder B. M. Ingersoll C. D. (2006). Reduced quadriceps activation after lumbar paraspinal fatiguing exercise. J. Athl. Train. 41:79. 10.1249/00005768-200505001-02178 16619099 PMC1421484

[B33] Hegedus E. J. McDonough S. Bleakley C. Cook C. E. Baxter G. D. (2015). Clinician-friendly lower extremity physical performance measures in athletes: a systematic review of measurement properties and correlation with injury, part 1. the tests for knee function including the hop tests. Br. J. Sports Med. 49, 642–648. 10.1136/bjsports-2014-094094 25497489

[B34] Helme M. Tee J. Emmonds S. Low C. (2021). Does lower-limb asymmetry increase injury risk in sport? A systematic review. Phys. Ther. Sport. 49, 204–213. 10.1016/j.ptsp.2021.03.001 33770741

[B35] Hogg J. A. Carlson L. M. Rogers A. Briles M. W. Acocello S. N. Wilkerson G. B. (2021). Reliability and concurrent validity of TRAZER compared to three-dimensional motion capture. J. Clin. Exp. Neuropsyc. 7:100. 10.18053/jctres.07.202101.013 34104813 PMC8177031

[B36] Hosmer D. W. Lemeshow S. (2000). Applied Logistic Regression. Hoboken, NJ: Wiley New York. 375p.

[B37] Hughes G. Musco P. Caine S. Howe L. (2020). Lower limb asymmetry after anterior cruciate ligament reconstruction in adolescent athletes: a systematic review and meta-analysis. J. Athl. Train. 55, 811–825. 10.4085/1062-6050-0244-19 32607546 PMC7462171

[B38] Huxel Bliven K. C. Anderson B. E. (2013). Core stability training for injury prevention. Sports Health 5, 514–522. 10.1177/1941738113481200 24427426 PMC3806175

[B39] Jensen A. R. (2006). Clocking the Mind: Mental Chronometry and Individual Differences. Oxford: Elsevier.

[B40] Kerr Z. Y. Wilkerson G. B. Caswell S. V. Currie D. W. Pierpoint L. A. Wasserman E. B.. (2018). The first decade of web-based sports injury surveillance: descriptive epidemiology of injuries in United States high school football (2005–2006 through 2013–2014) and National Collegiate Athletic Association football (2004–2005 through 2013–2014). J. Athl. Train. 53, 738–751. 10.4085/1062-6050-144-17 30138047 PMC6188086

[B41] King E. Richter C. Daniels K. A. Franklyn-Miller A. Falvey E. Myer G. D.. (2021). Biomechanical but not strength or performance measures differentiate male athletes who experience ACL reinjury on return to level 1 sports. Am. J. Sports Med. 49, 918–927. 10.1177/0363546520988018 33617291 PMC9677345

[B42] Kroshus E. Utter A. C. Pierpoint L. A. Currie D. W. Knowles S. B. Wasserman E. B.. (2018). The first decade of web-based sports injury surveillance: descriptive epidemiology of injuries in US high school boys' wrestling (2005–2006 through 2013–2014) and National Collegiate Athletic Association men's wrestling (2004–2005 through 2013–2014). J. Athl. Train. 53, 1143–1155. 10.4085/1062-6050-154-17 30721631 PMC6365066

[B43] Kuijpers T. van der Windt D. A. Boeke A. J. P. Twisk J. W. Vergouwe Y. Bouter L. M.. (2006). Clinical prediction rules for the prognosis of shoulder pain in general practice. Pain. 120, 276–285. 10.1016/j.pain.2005.11.004 16426760

[B44] Lambert C. Pfeiffer T. Lambert M. Brozat B. Lachmann D. Shafizadeh S.. (2020). Side differences regarding the limb symmetry index in healthy professional athletes. Int. J. Sports Med. 41, 729–735. 10.1055/a-1171-2548 32492733

[B45] Leetun D. T. Ireland M. L. Willson J. D. Ballantyne B. T. Davis I. M. (2004). Core stability measures as risk factors for lower extremity injury in athletes. Med. Sci. Sports Exerc. 36, 926–934. 10.1249/01.MSS.0000128145.75199.C3 15179160

[B46] Lempke L. B. Howell D. R. Eckner J. T. Lynall R. C. (2020). Examination of reaction time deficits following concussion: a systematic review and meta-analysis. Sports Med. 50, 1341–1359. 10.1007/s40279-020-01281-0 32162242

[B47] Lieberman D. E. Warrener A. G. Wang J. Castillo E. R. (2015). Effects of stride frequency and foot position at landing on braking force, hip torque, impact peak force and the metabolic cost of running in humans. J. Exp. Biol. 218, 3406–3414. 10.1242/jeb.125500 26538175

[B48] Luedke L. E. Geisthardt T. W. Rauh M. J. (2020). Y-balance test performance does not determine non-contact lower quadrant injury in collegiate American football players. Sports. 8:27. 10.3390/sports8030027 32120772 PMC7183065

[B49] Marques J. B. Paul D. J. Graham-Smith P. Read P. J. (2020). Change of direction assessment following anterior cruciate ligament reconstruction: a review of current practice and considerations to enhance practical application. Sports Med. 50, 55–72. 10.1007/s40279-019-01189-4 31531768 PMC6942029

[B50] McGovern R. P. Martin R. L. Christoforetti J. J. Kivlan B. R. (2018). Evidence-based procedures for performing the single leg squat and step-down tests in evaluation of non-arthritic hip pain: a literature review. Int. J. Sports Phys. Ther. 13:526. 10.26603/ijspt20180526 30038839 PMC6044589

[B51] Mertler C. A. Vannetta R. A. (2005). Advanced and Multivariate Statistical Methods. 3rd Edn. Los Angeles, CA: Pyrczak Publishing.

[B52] Moons K. G. Altman D. G. Reitsma J. B. Ioannidis J. P. Macaskill P. Steyerberg E. W.. (2015). Transparent reporting of a multivariable prediction model for individual prognosis or diagnosis (TRIPOD): explanation and elaboration. Ann. Intern. Med. 162, W1–W73. 10.7326/M14-0698 25560730

[B53] Moons K. G. de Groot J. A. Bouwmeester W. Vergouwe Y. Mallett S. Altman D. G.. (2014). Critical appraisal and data extraction for systematic reviews of prediction modelling studies: the CHARMS checklist. PLoS Med. 11:e1001744. 10.1371/journal.pmed.1001744 25314315 PMC4196729

[B54] Moran R. W. Schneiders A. G. Mason J. Sullivan S. J. (2017). Do functional movement screen (FMS) composite scores predict subsequent injury? a systematic review with meta-analysis. Br. J. Sports Med. 51, 1661–1669. 10.1136/bjsports-2016-096938 28360142

[B55] Ness B. M. Zimney K. Schweinle W. E. Cleland J. A. (2020). Dual-task assessment implications for anterior cruciate ligament injury: a systematic review. Int. J. Sports Phys. Ther. 15:840. 10.26603/ijspt20200840 33344002 PMC7727432

[B56] Nuzzo J. L. (2015). The National Football League scouting combine from 1999 to 2014: normative reference values and an examination of body mass normalization techniques. J. Strength Cond. Res. 29, 279–289. 10.1519/JSC.0000000000000755 25436631

[B57] Paap K. R. Sawi O. (2016). The role of test-retest reliability in measuring individual and group differences in executive functioning. J. Neurosci. Methods 274, 81–93. 10.1016/j.jneumeth.2016.10.002 27720867

[B58] Panken G. Verhagen A. P. Terwee C. B. Heymans M. W. (2017). Clinical prediction models for patients with nontraumatic knee pain in primary care: a systematic review and internal validation study. J. Orthop. Sports Phys. Ther. 47, 518–529. 10.2519/jospt.2017.7142 28622751

[B59] Park K.-M. Cynn H.-S. Choung S.-D. (2013). Musculoskeletal predictors of movement quality for the forward step-down test in asymptomatic women. J. Orthop. Sports Phys. Ther. 43, 504–510. 10.2519/jospt.2013.4073 23756380

[B60] Pavlou M. Ambler G. Seaman S. De Iorio M. Omar R. Z. (2016). Review and evaluation of penalised regression methods for risk prediction in low-dimensional data with few events. Stat. Med. 35, 1159–1177. 10.1002/sim.6782 26514699 PMC4982098

[B61] Prieto-Marañón P. Aguerri M. E. Galibert M. S. Attorresi H. F. (2012). Detection of differential item functioning: using decision rules based on the Mantel-Haenszel procedure and Breslow-Day tests. Methodol. Eur. J. Res. Meth. Behav. Soc. Sci. 8:63. 10.1027/1614-2241/a000038

[B62] Read P. J. McAuliffe S. Bishop C. Oliver J. L. Graham-Smith P. Farooq M. A. (2021). Asymmetry thresholds for common screening tests and their effects on jump performance in professional soccer players. J. Athl. Train. 56, 46–53. 10.4085/1062-6050-0013.20 33264407 PMC7863609

[B63] Schiltz M. Lehance C. Maquet D. Bury T. Crielaard J.-M. Croisier J.-L. (2009). Explosive strength imbalances in professional basketball players. J. Athl. Train. 44, 39–47. 10.4085/1062-6050-44.1.39 19180217 PMC2629038

[B64] Serrien D. J. Ivry R. B. Swinnen S. P. (2006). Dynamics of hemispheric specialization and integration in the context of motor control. Nat. Rev. Neurosci. 7, 160–166. 10.1038/nrn1849 16429125

[B65] Soe M. M. Sullivan K. M. Dean A. G. (2005). OpenEpi: Open Source Epidemiologic Statistics for Public Health: Power for Cohort Studies. Atlanta, GA: OpenEpi. Available online at: http://www.openepi.com/Power/PowerCohort.htm (accessed March 1, 2013).

[B66] Tabachnick B. G. Fidell L. S. Ullman J. B. (2007). Using Multivariate Statistics. Boston, MA: Pearson.

[B67] Takeuchi N. Oouchida Y. Izumi S.-I. (2012). Motor control and neural plasticity through interhemispheric interactions. Neural Plast. 2012:823285. 10.1155/2012/823285 23326685 PMC3541646

[B68] Tarara D. T. Fogaca L. K. Taylor J. B. Hegedus E. J. (2016). Clinician-friendly physical performance tests in athletes part 3: a systematic review of measurement properties and correlations to injury for tests in the upper extremity. Br. J. Sports Med. 50, 545–551. 10.1136/bjsports-2015-095198 26701926

[B69] Teyhen D. S. Flynn T. W. Childs J. D. Abraham L. D. (2007). Arthrokinematics in a subgroup of patients likely to benefit from a lumbar stabilization exercise program. Phys. Ther. 87, 313–325. 10.2522/ptj.20060253 17311885

[B70] Teyhen D. S. Shaffer S. W. Goffar S. L. Kiesel K. Butler R. J. Rhon D. I.. (2020). Identification of risk factors prospectively associated with musculoskeletal injury in a warrior athlete population. Sports Health 12, 564–572. 10.1177/1941738120902991 32134698 PMC7785899

[B71] Vereijken A. Aerts I. Jetten J. Tassignon B. Verschueren J. Meeusen R.. (2020). Association between functional performance and return to performance in high-impact sports after lower extremity injury: a systematic review. J. Sci. Med. Sport. 19:564–576. 32874110 PMC7429422

[B72] Wellman A. D. Coad S. C. Goulet G. C. McLellan C. P. (2017). Quantification of accelerometer derived impacts associated with competitive games in National Collegiate Athletic Association Division I college football players. J. Strength Cond. Res. 31, 330–338. 10.1519/JSC.0000000000001506 27227790

[B73] Wilkerson G. B. Acocello S. N. Davis M. B. Ramos J. M. Rucker A. J. Hogg J. A. (2021b). Wellness survey responses and smartphone app response efficiency: associations with remote history of sport-related concussion. Percept. Mot. Skills. 128, 714–730. 10.1177/0031512520983680 33357092

[B74] Wilkerson G. B. Colston M. A. (2015). A refined prediction model for core and lower extremity sprains and strains among collegiate football players. J. Athl. Train. 50, 643–650. 10.4085/1062-6050-50.2.04 25844856 PMC4527449

[B75] Wilkerson G. B. Giles J. L. Seibel D. K. (2012). Prediction of core and lower extremity strains and sprains in collegiate football players: a preliminary study. J. Athl. Train. 47, 264–272. 10.4085/1062-6050-47.3.17 22892407 PMC3392156

[B76] Wilkerson G. B. Grooms D. R. Acocello S. N. (2017). Neuromechanical considerations for post-concussion musculoskeletal injury risk management. Curr. Sports Med. Rep. 16, 419–427. 10.1249/JSR.0000000000000430 29135640

[B77] Wilkerson G. B. Nabhan D. C. Crane R. T. (2020). Concussion history and neuromechanical responsiveness asymmetry. J. Athl. Train. 55, 594–600. 10.4085/1062-6050-0401.19 32396473 PMC7319744

[B78] Wilkerson G. B. Nabhan D. C. Crane R. T. (2021a). Upper-extremity perceptual-motor training improves whole-body reactive agility among elite athletes with history of sport-related concussion. J. Sport Rehabil. 1, 1–6. 10.1123/jsr.2020-0337 33418536

[B79] Wilkerson G. B. Nabhan D. C. Prusmack C. J. Moreau W. J. (2018). Detection of persisting concussion effects on neuromechanical responsiveness. Med. Sci. Sports Exerc. 50, 1750–1756. 10.1249/MSS.0000000000001647 29683918

[B80] Wingerson M. J. Seehusen C. N. Walker G. Wilson J. C. Howell D. R. (2020). Clinical feasibility and utility of a dual-task tandem gait protocol for pediatric concussion management. J Athl Train. 10.4085/323-20 33150416 PMC10072090

[B81] Youden W. J. (1950). Index for rating diagnostic tests. Cancer. 3, 32–35. 10.1002/1097-0142(1950)3:1<32::AID-CNCR2820030106>3.0.CO;2-3 15405679

[B82] Zazulak B. T. Hewett T. E. Reeves N. P. Goldberg B. Cholewicki J. (2007). Deficits in neuromuscular control of the trunk predict knee injury risk: prospective biomechanical-epidemiologic study. Am. J. Sports Med. 35, 1123–1130. 10.1177/0363546507301585 17468378

