# Supplementary material for: Whole-Body Reactive Agility Metrics to Identify Football Players With a Core and Lower Extremity Injury Risk
Source: Front Sports Act Living. 2021 Oct 20;3:733567. doi: 10.3389/fspor.2021.733567 (PMC8564038; doi:10.3389/fspor.2021.733567)
Supplement: Supplementary file 1 [file Data_Sheet_1.PDF]

Histogram of the distribution of the DA RT Avg times

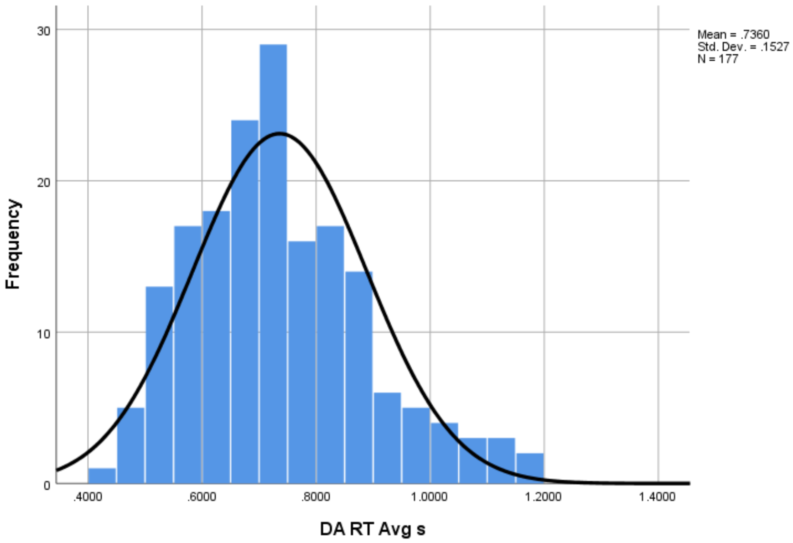

Histogram of the distribution of the DA RT Avg times for those participants who suffered a CLEI vs. those who did not suffer a CLEI

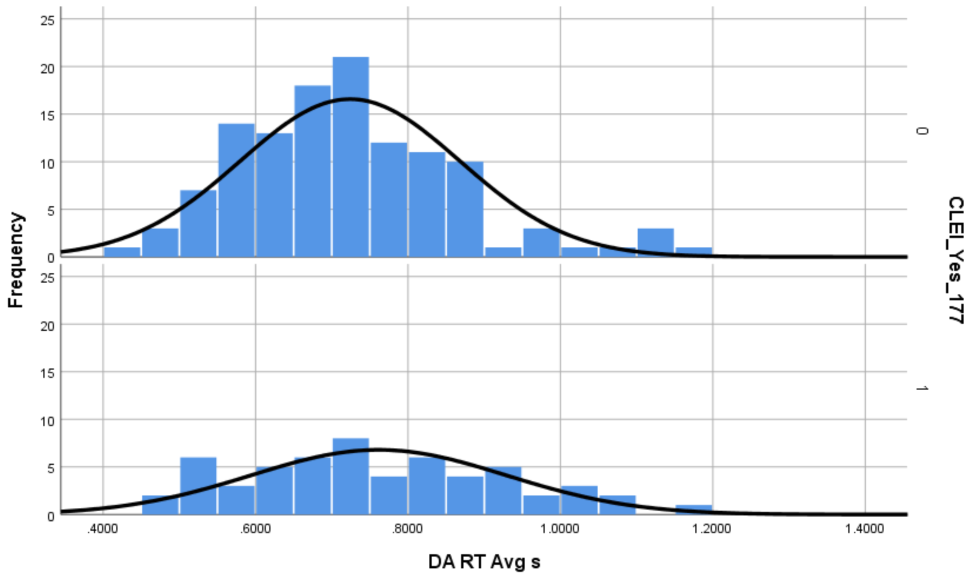

Not Injured

Injured

Histogram of the distribution of the log 10 transformed for DA RT Avg times

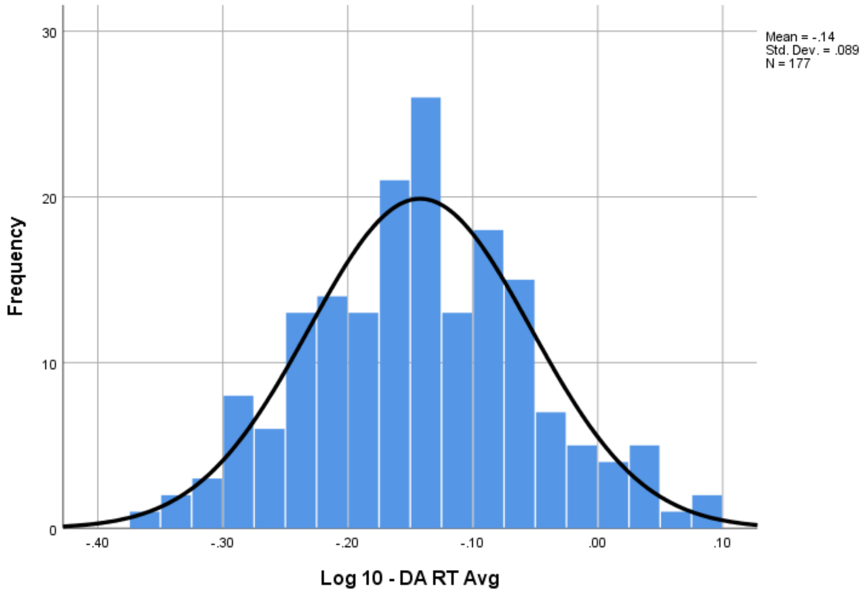

Histogram of the distribution of the log 10 transformed for DA RT Avg times for those participants who suffered a CLEI vs. those who did not suffer a CLEI

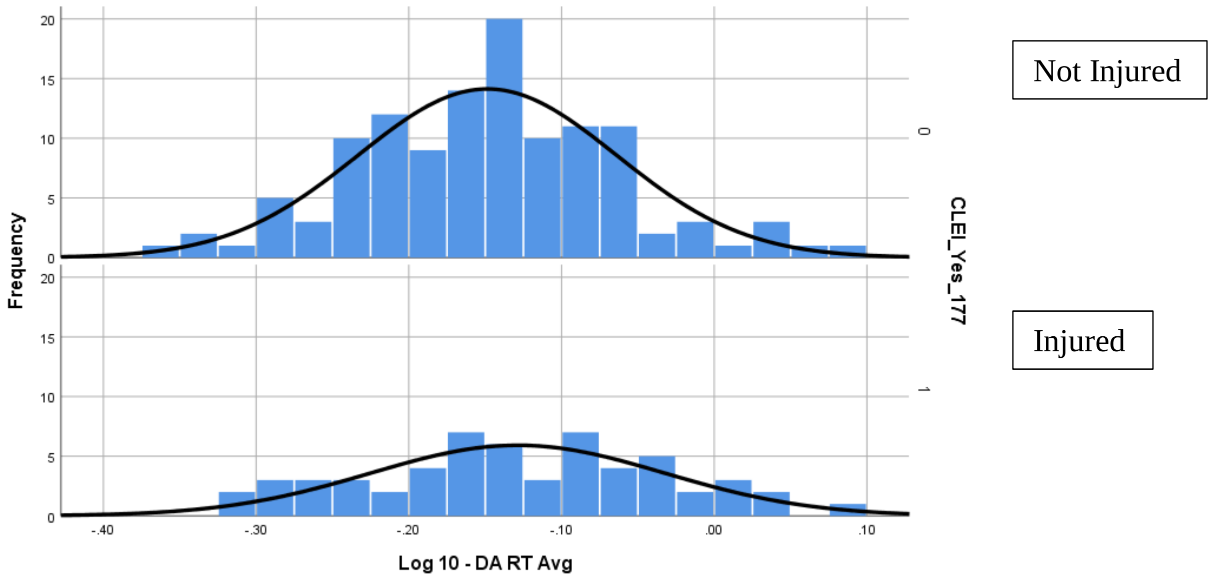

**Mean RT Avg** (N = 177)				
	**Minimum**	**Maximum**	**Mean**	**Std. Deviation**
LA RT Avg s	0.245	0.970	0.561	0.131
LF RT Avg s	0.739	1.98	1.09	0.264
DA RT Avg s	0.444	1.20	0.736	0.153
DF RT Avg s	0.731	1.74	1.07	0.205

Sum of the 3 factors x CLEI

		Injured	No Injured	Total
< 2 Factors	Count	21	92	113
	% within < 2 Factors	18.6%	81.4%	100.0%
≥ 2 Factors	Count	36	28	64
	% within ≥ 2 Factors	56.3%	43.7%	100.0%
Total	Count	57	120	177
	% within Sum of 3 Factors	32.2%	67.8%	100.0%

$\chi^2 = 26.81, p < 0.001$
